# Thermographic Fault Diagnosis of Shaft of BLDC Motor

**DOI:** 10.3390/s22218537

**Published:** 2022-11-05

**Authors:** Adam Glowacz

**Affiliations:** Department of Automatic Control and Robotics, Faculty of Electrical Engineering, Automatics, Computer Science and Biomedical Engineering, AGH University of Science and Technology, al. A. Mickiewicza 30, 30-059 Kraków, Poland; adglow@agh.edu.pl

**Keywords:** thermal, image, fault, diagnosis, neural network

## Abstract

A technique of thermographic fault diagnosis of the shaft of a BLDC (Brushless Direct Current Electric) motor is presented in this article. The technique works for the shivering of the thermal imaging camera in the range of 0–1.5 [m/s^2^]. An electric shaver was used as the source of the BLDC motor. The following states of the BLDC motor were analyzed: Healthy BLDC motor (HB), BLDC motor with one faulty shaft (1FSB), BLDC motor with two faulty shafts (2FSB), and BLDC motor with three faulty shafts (3FSB). A new method of feature extraction named PNID (power of normalized image difference) was presented. Deep neural networks were used for the analysis of thermal images of the faulty shaft of the BLDC motor: GoogLeNet, ResNet50, and EfficientNet-b0. The results of the proposed technique were very good. PNID, GoogLeNet, ResNet50, and EfficientNet-b0 have an efficiency of recognition equal to 100% for four classes.

## 1. Introduction

Electric motors are used in many areas of industry, for example, mining and oil industries. Electric motors differ in their design, operating principle, and power. They can be used for various applications: Washing machines, dusters, mixers, industrial machines, cars, and trains. In many areas, it would be difficult for us to live without motors. Some examples of where the electric motor is used are as follows: In cars as a starter, escalators, production lines, lathes, and grinders, and even in home appliances such as printers and electric toothbrushes. Electric motors do not use any fuel. They do not emit exhaust or other gases. Therefore, they can work in closed halls, garages, and even in very small spaces. Moreover, electric motors are produced in sealed housings. Thus, they do not generate sparks, meaning they are useful in hazardous areas.

Electric motors are not immune to malfunctions and gradual deterioration. They need regular maintenance to avoid motor failures. Fault detection of electric motors is essential to reduce maintenance costs. The maintenance costs of electric motors can be high if the machines are not diagnosed on time. Faulty electric motors draw additional electricity to operate. Permanently damaged motors should be replaced with new ones. Replacing an electric motor is expensive. Moreover, the failure of an electric motor during operation can stop the production line in the factory. This is a financial and energy loss for the factory. Fault diagnosis of motors can improve their performance. With continuous use, electric motors slowly degrade over time. An increase in the equipment lifespan of a motor can be also performed using fault detection techniques.

Fault diagnostics of electric motors include various faults, e.g., a faulty shaft, faulty bearings, faulty gear, a faulty fan, an imbalance of the shaft, broken rotor bars, and shorted coils. A technique of thermographic fault diagnosis of the shaft of a BLDC (Brushless Direct Current Electric) motor is presented in this article. A faulty shaft can cause an imbalance. Imbalance causes premature failure in electrical motors. It usually causes noise, heat, and vibration. If the damaged shaft is not repaired, the motor will be damaged again. The author’s motivation is to develop new methods of diagnosing electric motors.

The main contribution of the paper is the implementation and analysis of the technique of thermographic fault diagnosis of the shaft of the BLDC motor. The technique works for the shivering of the thermal imaging camera in the range of 0–1.5 [m/s^2^]. The following states of BLDC motors were analyzed: HB, 1FSB, 2FSB, and 3FSB. PNID (power of normalized image difference), GoogLeNet, ResNet50, and EfficientNet-b0 were used for the analysis of thermal images of the faulty shaft of the BLDC motor. The obtained efficiency *E* and *MeanE* were equal to 100%. PNID, GoogLeNet, ResNet50, and EfficientNet-b0 work very well for the analysis of thermal images. Verification was carried out using GoogLeNet, ResNet50, and EfficientNet-b0. A new method of feature extraction, PNID (power of normalized image difference), was presented. Deep neural networks were used for the analysis of thermal images of the faulty shaft of the BLDC motor, namely, GoogLeNet, ResNet50, and EfficientNet-b0.

The article consists of six sections: Introduction, measurements, related works, thermographic fault diagnosis technique, results, and conclusions.

**Figure 1:**
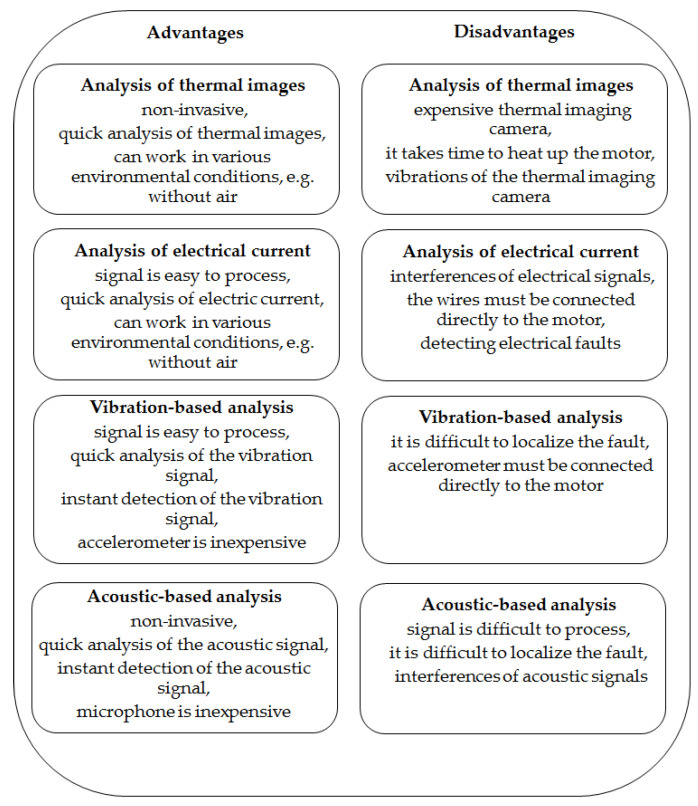
Advantages and disadvantages of fault diagnosis techniques.

In the literature, there are several fault diagnosis techniques: Acoustic-based analysis, vibration-based analysis, analysis of electrical current, and analysis of thermal images. The advantages and disadvantages of fault diagnosis techniques are presented in Figure 1. The major benefits are as follows: The non-invasive measurement, due to which it can be used for many types of faults and applications; electrical and mechanical faults can also be analyzed; it is safe for the operator, and it can also be used for fault diagnosis of expensive planes and spacecraft.

The analysis of thermal images is presented in the literature [1,2,3,4,5,6,7,8]. Worm gear condition monitoring using thermal images was presented in reference [1]. Infrared thermal images were analyzed using a convolutional neural network. The accuracy of fault diagnosis of healthy and faulty gearboxes was equal to 100% for the IRT-CNN method. A methodology for fault diagnosis of a kinematic chain using thermal images was described [2]. The following conditions were analyzed: Broken rotor bars, faulty bearing, healthy, unbalanced, misalignment, and wearing of the gearbox. Principal Component Analysis and the artificial neural network were used for the analysis. The accuracy of the proposed technique was equal to 96.8%. The fatigue behavior of mechanical components using thermal thermography has been discussed in the literature [3]. The results of the thermographic technique were close to the staircase method. The described procedure is viable and fast for fatigue tests. It can be used for complex structural components such as crankshafts. Worm gearbox fault diagnosis using vibrations, sounds, and thermal images was presented in the work [4]. The following states of the worm gearbox were analyzed: Pitting, tooth breakage, healthy, and wear. Artificial neural networks and support vector machines were used for the analysis. Vibrations, sounds, and thermal images were used for classification. The accuracy of the analysis was 99.2% for artificial neural networks and 98.7% for support vector machines. Eddy current pulsed thermography was described in a previous paper [5]. It was a nondestructive testing technology. It can be used for conductive metal materials and selected non-metallic materials. The framework for rotating machinery fault diagnosis was described in the literature [6]. Vibration signals, infrared images, and a confidence weight support matrix machine were used for the analysis. Average classification accuracies still exceeded 97%. A fault diagnosis of rotating machinery was shown in reference [7]. Ten states of various speeds (900–3000 rpm) were analyzed. The analysis was carried out with infrared images and the least-squares interactive support matrix machine. The average accuracy of the analysis exceeded 98%. Fault diagnosis of rotating machinery was described in reference [8]. Infrared images and vibration signals were used for the fault diagnosis of the rotor-bearing system. A convolutional neural network was used for the analysis of signals. The average accuracy of the proposed technique was equal to 98%.

The analysis of the electrical current of electric motors was presented in references [9,10]. In a previous paper, motor stator currents and vibration signals were used for the detection of faults in induction motors [9]. The states of induction motors were the following: Healthy condition, unbalanced condition of shaft rotation, bearing fault, combined bearing fault and one broken rotor bar, and unbalance voltage. Feature extraction was based on matching pursuit and discrete wavelet transform. Eight features were used for training and testing. The classification was carried out using SVM, KNN, and Bagged Trees (mostly 100% accuracy). Incipient winding fault detection of induction motors was presented in reference [10]. The proposed method was based on electrical current analysis. Turn-to-turn short faults and broken rotor bars were analyzed. The computed results showed that the described method provides the proper diagnosis of faults.

Vibration-based analysis was described in references [11,12]. The authors proposed PCA and the Bayesian sensor fusion method for fault diagnosis of induction motors in the literature [11]. Current, acoustic, and vibration signals were analyzed. The authors analyzed bearing faults stator faults and broken rotor bar faults. The results of the analysis were in the range of 96.15–99.96%. Fault diagnosis using hybrid weighted deep adversarial learning was described in reference [12]. Experiments were carried out for two rotating-machine datasets. Vibration signals were analyzed. The sampling frequency of the accelerometer was equal to 5 kHz. The following states were analyzed: Healthy, rolling fault, inner race fault, and outer race fault. The proposed approach was very good. Computed accuracies were higher than 70%. It can be used for industrial applications.

Acoustic-based analysis was presented in references [13,14]. An acoustic analysis of three electric impact drills and angle grinders was presented in reference [13]. Three states of the electric impact drill and three states of the angle grinder were analyzed. Analysis was carried out using SMOFS-NFC, the nearest neighbor classifier, and the Naive Bayes classifier. The computed accuracy was in the range of 89.33–97.33% for electric impact drills and 90.66–100% for the three angle grinders. An approach based on deep learning for bearing fault diagnosis was presented in reference [14]. Acoustic signals were analyzed using STFT. Next, computed subpatterns were used by the LAMSTAR (large memory storage retrieval) network and CNN. The authors of the paper analyzed ball faults, outer race faults, inner race faults, cage faults, and healthy condition. LAMSTAR’s overall accuracy was in the range of 96–100%.

## 2. Measurements

**Figure 2:**
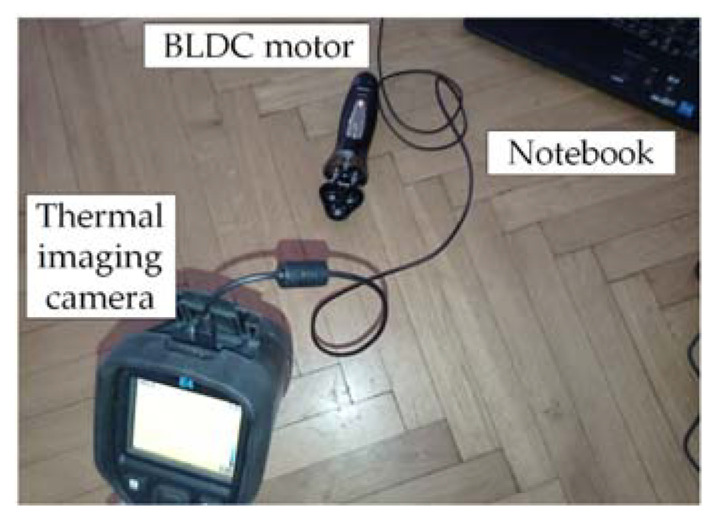
Proposed experimental setup.

The proposed experimental setup (thermal imaging camera, computer, and implemented fault diagnosis technique) was placed in a room (Figure 2).

**Figure 3:**
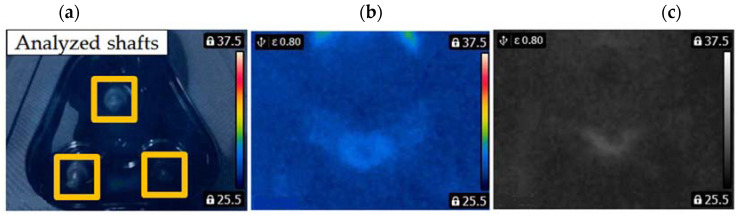
(HB) (**a**) Iron scale; (**b**) rainbow scale; (**c**) gray-scale.

**Figure 4:**
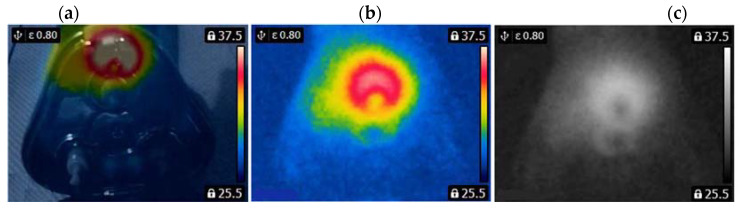
(1FSB) (**a**) Iron scale; (**b**) rainbow scale; (**c**) gray-scale.

**Figure 5:**
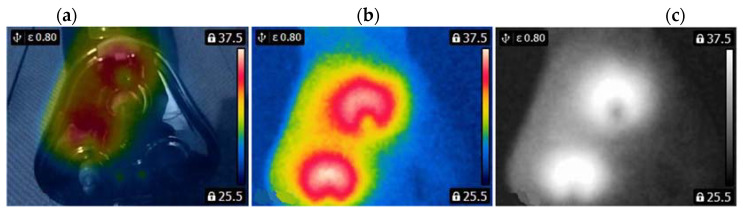
(2FSB) (**a**) Iron scale; (**b**) rainbow scale; (**c**) gray-scale.

**Figure 6:**
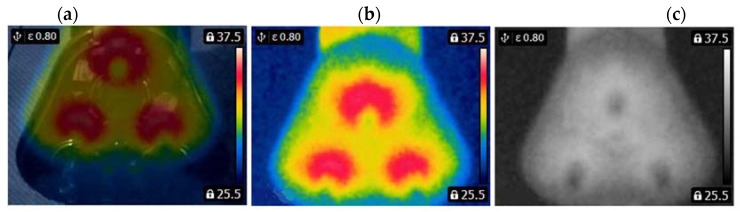
(3FSB) (**a**) Iron scale; (**b**) rainbow scale; (**c**) gray-scale.

A FLIR E4 camera was used to capture thermal images of the BLDC motor. The distance of the thermal camera to the BLDC motor was equal to 0.5 [m]. The shivering of the thermal imaging camera was in the range of 0–1.5 [m/s^2^]. The obtained thermal images were different. Thermal images were measured for an emissivity coefficient ε = 0.8. The emissivity coefficient ε = 0.8 was accurate for analyzed thermal images (Figure 3, Figure 4, Figure 5 and Figure 6). White objects were clearly visible (Figure 3c, Figure 4c, Figure 5c and Figure 6c).

BLDC motors of electric shavers were used (Prime3 Men’s Shaver SRS11, Philips S5587/30, and Philips S1332/41). The analyzed electric shavers were similar to each other. The parameters of the Prime3 Men’s Shaver SRS11 electric shaver were *V* = 230 V (voltage), *F* = 50 Hz (frequency), *I* = 0.2 A (electric current), *M* = 0.19 kg (weight), and very quiet operation—up to 48 dB. The parameters of the Philips S5587/30 electric shaver were *V_p_* = 100–240 V (voltage), *F_p_* = 50 Hz (frequency), *I_p_* = 0.36 A (electric current), *P_p_* = 9 W (power of the motor), and *M_p_* = 0.21 kg. The parameters of the Philips S1332/41 electric shaver were *V_s_* = 100–240 V (voltage), *F_s_* = 50 Hz (frequency), *I_s_*= 0.36 A (electric current), *P_s_* = 9 W (power of the motor), and *M_s_* = 0.19 kg.

The following states of BLDC were analyzed: Healthy BLDC motor (HB), BLDC motor with 1 faulty shaft (1FSB), BLDC motor with 2 faulty shafts (2FSB), and BLDC motor with 3 faulty shafts (3FSB). Thermal images of the analyzed states of the BLDC motor (Prime3 Men’s Shaver SRS11) are presented (Figure 3, Figure 4, Figure 5 and Figure 6). The thermal images of a healthy BLDC motor (HB) are shown in Figure 3.

The thermal images of the BLDC motor with 1 faulty shaft (1FSB) are shown in Figure 4.

The thermal images of the BLDC motor with 2 faulty shafts (2FSB) are shown in Figure 5.

The thermal images of the BLDC motor with 3 faulty shafts (3FSB) are shown in Figure 6.

State HB has a large number of cold areas (blue/gray color). State 3FSB has a large number of hot areas (red/white color).

**Figure 7:**
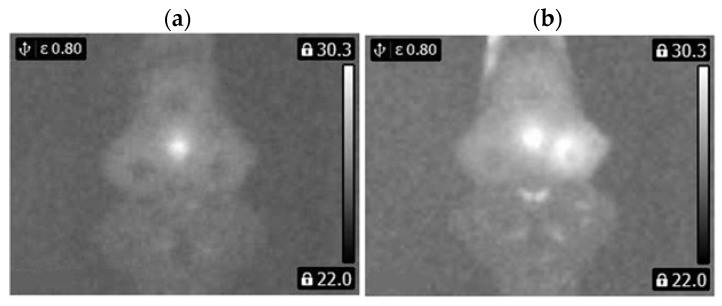
(**a**) Gray-scale thermal image of HB state, (**b**) gray-scale thermal image of 1FSB state (Philips S5587/30).

**Figure 8:**
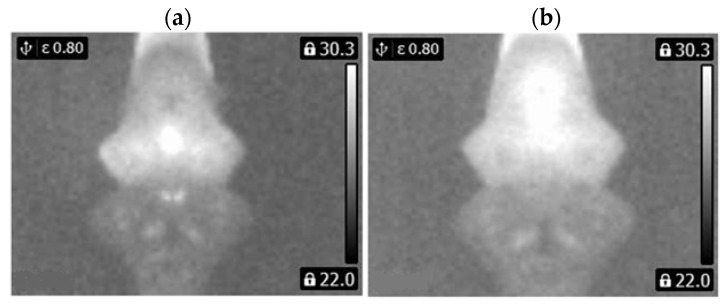
(**a**) Gray-scale thermal image of 2FSB state, (**b**) gray-scale thermal image of 3FSB state (Philips S5587/30).

Thermal images of the analyzed states of the BLDC motor (Philips S5587/30) are presented (Figure 7 and Figure 8).

**Figure 9:**
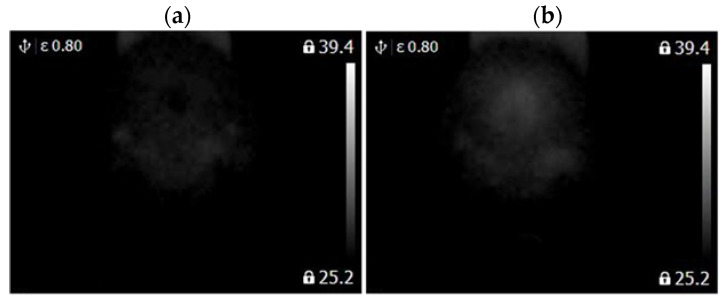
(**a**) Gray-scale thermal image of HB state, (**b**) gray-scale thermal image of 1FSB state (Philips S1332/41).

**Figure 10:**
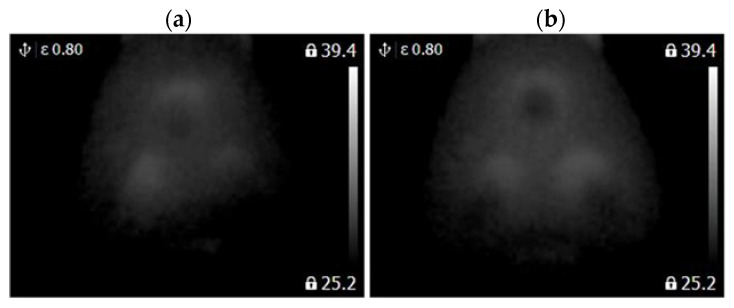
(**a**) Gray-scale thermal image of 2FSB state, (**b**) gray-scale thermal image of 3FSB state (Philips S1332/41).

Thermal images of the analyzed states of the BLDC motor (Philips S1332/41) are presented (Figure 9 and Figure 10).

Measurements of the BLDC motor states were carried out for operating motors. The temperature of the motors after 5 min of operation was similar to that of a few hours. These motors do not heat up much. For motors with higher powers, measurements should be made after 1 h of operation. A faulty electric motor gives a higher surface temperature. The higher surface temperature is detected and analyzed using the proposed fault diagnosis technique.

## 3. Thermographic Fault Diagnosis Technique

**Figure 11:**
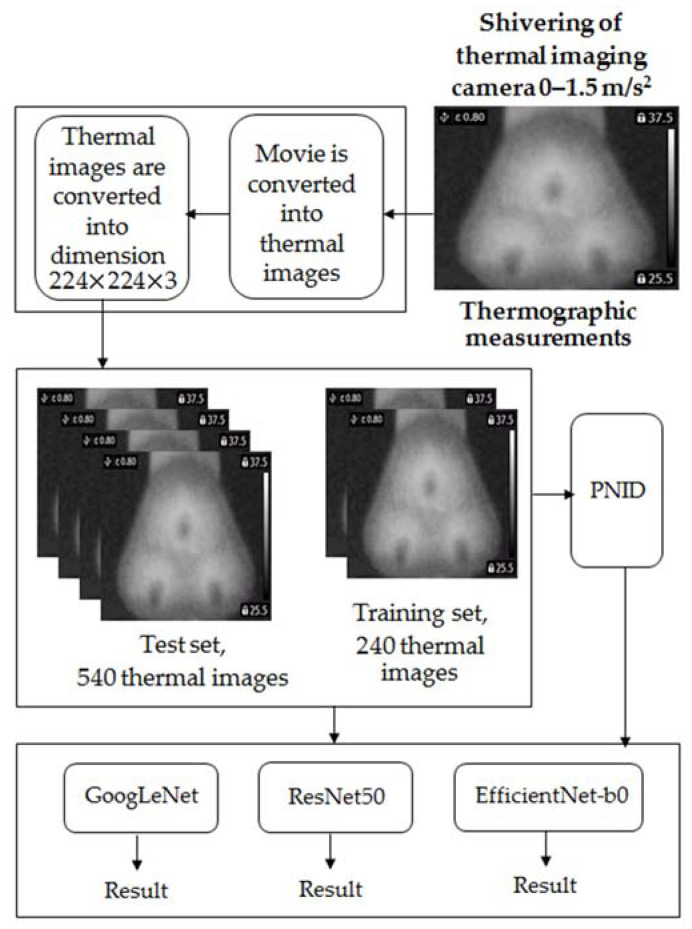
Thermographic fault diagnosis of the BLDC motor.

The thermographic fault diagnosis technique consists of six steps of image processing: Thermographic measurement, conversion of the movie into thermal images, conversion of thermal images into the proper dimensions of 224 × 224 × 3, feature extraction using PNID (power of normalized image difference), training of the deep neural network, testing of the deep neural network, and additional verification using the deep neural network (Figure 11). The movie was captured using a FLIR E4 thermal imaging camera. The captured movie was converted into thermal images. Next, thermal images were converted into the proper dimensions of 224 × 224 × 3. Dimensions of 224 × 224 × 3 were required for GoogLeNet, ResNet50, and EfficientNet-b0. Feature extraction was carried out using PNID. Training of the deep neural network was performed for training samples (240 training thermal images, 60 for each class). Testing of the deep neural network was performed for test samples (720 test thermal images, 180 for each class). Verification was carried out using GoogLeNet, ResNet50, and EfficientNet-b0. Matlab software was used for the implementation of the proposed fault diagnosis technique.

### 3.1. PNID (Power of Normalized Image Difference)

The new method of feature extraction—PNID (power of normalized image difference)—is presented. The PNID method has the following steps:

1. Create training and test sets.
2. Compute differences of thermal images: **diff_hb_1f**_1_ = |**hb**_1_**-1f**_1_|,…, **diff_hb_1f**_30_ = |**hb**_30_**-1f**_30_|, **diff_hb_2f**_1_ = |**hb**_1_**-2f**_1_|,…, **diff_hb_3f**_1_ = |**hb**_1_**-3f**_1_|,…, **diff_1f_2f**_1_ = |**1f**_1_**-2f**_1_|,…, **diff_1f_3f**_1_ = |**1f**_1_**-3f**_1_|,…, **diff_2f_3f**_1_ = |**2f**_1_**-3f**_1_|,…, **diff_2f_3f**_30_ = |**2f**_30_**-3f**_30_|; **hb**—matrix of thermal image of class HB, **1f**—matrix of thermal image of class 1FSB, **2f**—matrix of thermal image of class 2FSB, **3f**—matrix of thermal image of class 3FSB.
3. Compute matrix **R**, where **R** = **diff_hb_1f**_1_ + **…** + **diff_hb_1f**_30_ + **diff_hb_2f**_1_ + **…** + **diff_hb_2f**_30_ + **diff_hb_3f**_1_ + **…** + **diff_hb_3f**_30_ + **diff_1f_2f**_1_ + **…** + **diff_1f_2f**_30_ + **diff_1f_3f**_1_ + **…** + **diff_1f_3f**_30_ + **diff_2f_3f**_1_ + **…** + **diff_2f_3f**_30_.
4. Compute *R_max_*—maximum value of matrix **R**.
5. Compute matrix **M** = **R**/*R_max_*.
6. Compute the value *B*ij = *M*_ij_ × *M*_ij_, *B*_ij_—new value of matrix **B**, *M*_ij_—the value of matrix **M**.
7. Compute matrix **B**.
8. Compute the *t* threshold of binarization using Otsu’s method for matrix **B**. Compute the power of the computed threshold of binarization *t^2^*.
9. Create binarization of matrix **B** using a new level of binarization (*t*^2^). Compute matrix **A**.
10. Compute **N** = **S** + **A**, where **S**—matrix of the analyzed thermal image.
11. Compute **V**=**N**−1.
12. Change values less than 0 to 0 for matrix **V**. Form the output matrix **Z** (analyzed feature).

**Figure 12:**
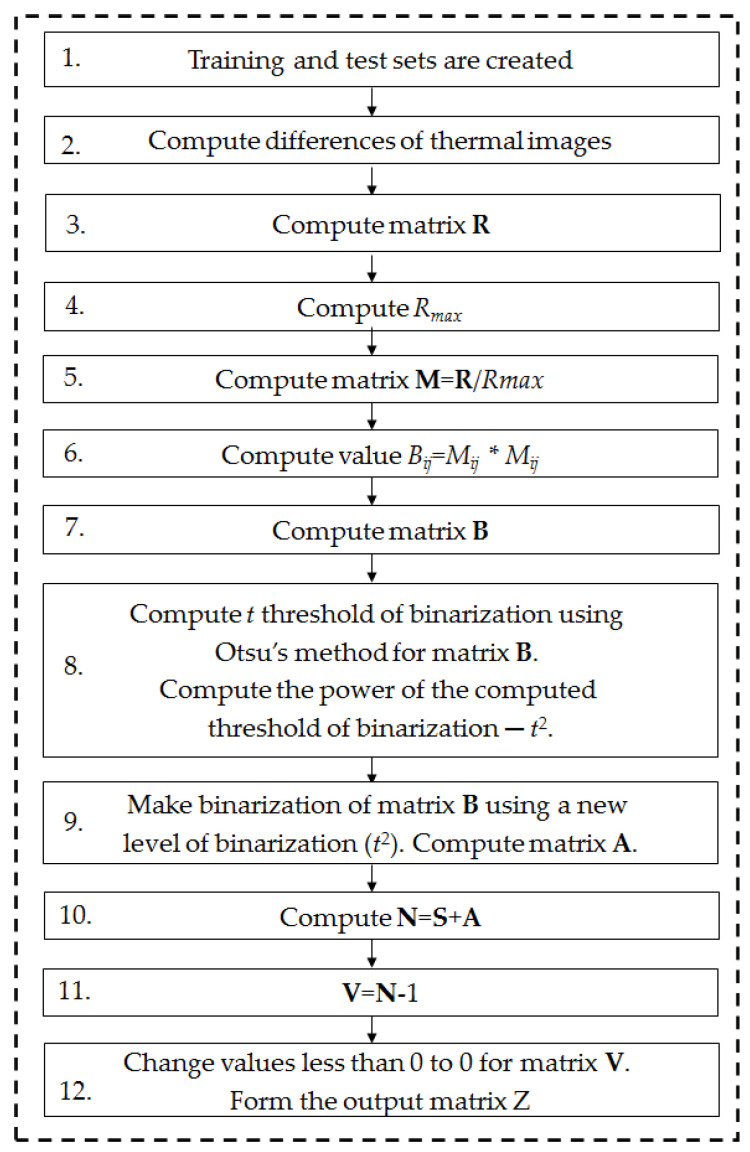
Flowchart of the PNID method.

A flowchart of the PNID method is depicted in Figure 12.

**Figure 13:**
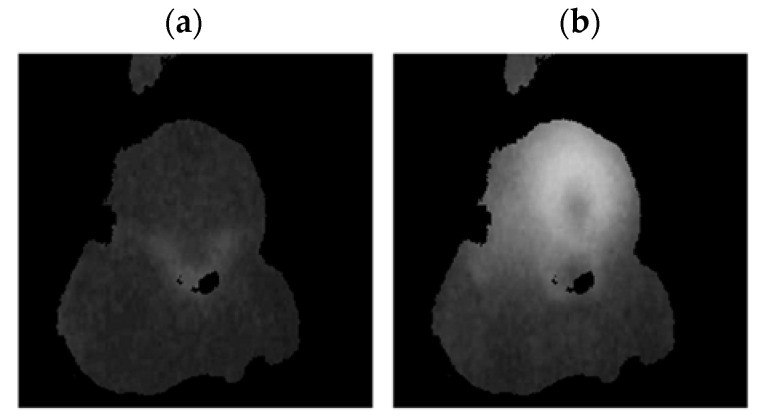
Matrix **Z** for PNID method: (**a**) HB, (**b**) 1FSB (Prime3 Men’s Shaver SRS11).

**Figure 14:**
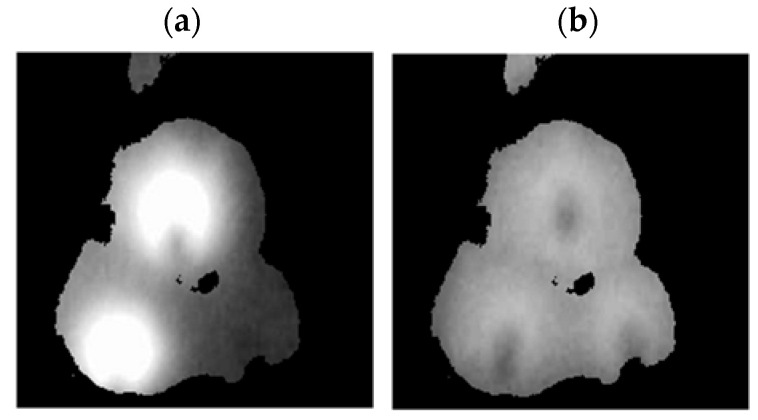
Matrix **Z** for PNID method: (**a**) 2FSB, (**b**) 3FSB (Prime3 Men’s Shaver SRS11).

Matrices **Z** of the analyzed states for the PNID method are presented in Figure 13 and Figure 14 (Prime3 Men’s Shaver SRS11). The computed threshold of binarization *t* (step 8 in Figure 12) was equal to 0.3686 for the analyzed thermal images.

**Figure 15:**
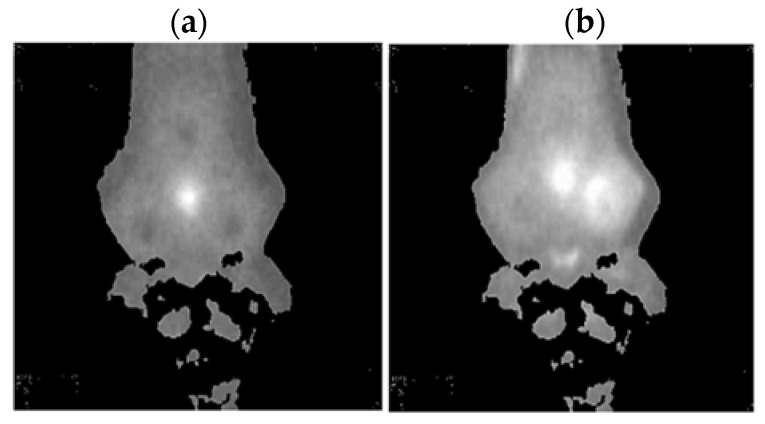
Matrix **Z** for PNID method: (**a**) HB, (**b**) 1FSB (Philips S5587/30).

**Figure 16:**
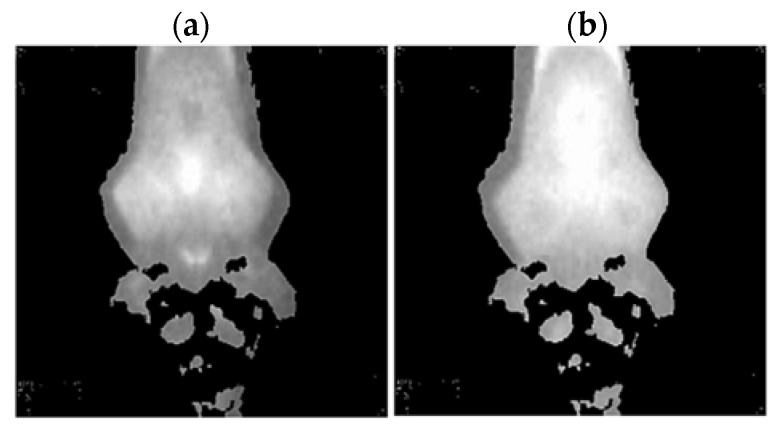
Matrix **Z** for PNID method: (**a**) 2FSB, (**b**) 3FSB (Philips S5587/30).

Matrices **Z** of the analyzed states for the PNID method are presented in Figure 15 and Figure 16 (Philips S5587/30). The computed threshold of binarization *t* was equal to 0.2235 for the analyzed thermal images.

**Figure 17:**
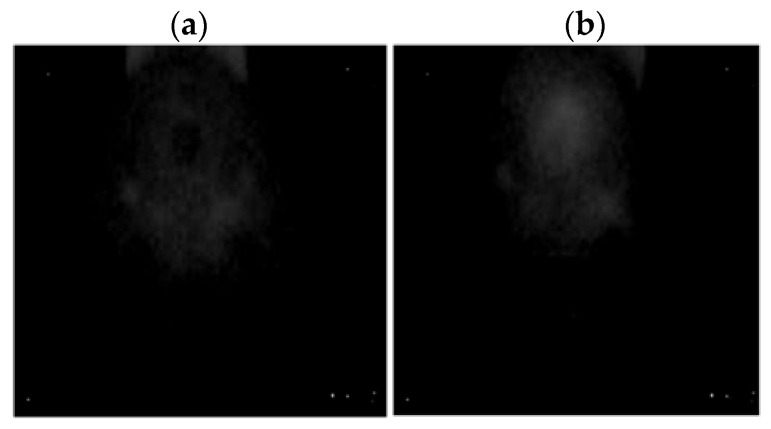
Matrix **Z** for PNID method: (**a**) HB, (**b**) 1FSB (Philips S1332/41).

**Figure 18:**
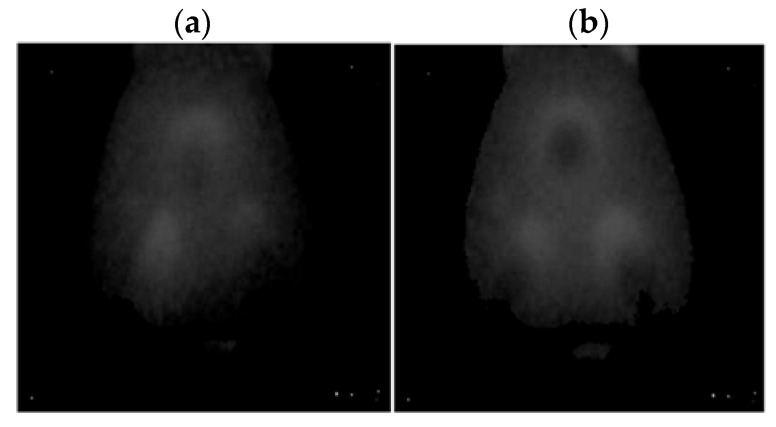
Matrix **Z** for PNID method: (**a**) 2FSB, (**b**) 3FSB (Philips S1332/41).

Matrices **Z** of the analyzed states for the PNID method are presented in Figure 17 and Figure 18 (Philips S1332/41). The computed threshold of binarization *t* was equal to 0.2196 for the analyzed thermal images.

The computed areas are well recognizable. We can note white areas for faulty states of the BLDC motor. Computed matrices **Z** are used as the input for convolutional neural networks.

### 3.2. Convolutional Neural Network (CNN)

Neural networks are used for fault diagnosis, the verification of technical equipment, image recognition, and industrial fault diagnosis systems [15,16,17,18]. There are several types of Convolutional Neural Networks (CNNs): GoogLeNet [19,20,21], ResNet50 [22,23,24], and EfficientNet-b0 [25,26,27]. CNNs can assign importance to objects in the thermal image. Next, it can recognize one image from another. The visual cortex of animals was the inspiration for the organization of CNNs. CNNs consist of three types of layers: Convolutional, pooling, and fully connected layers. The application of CNNs can be found in face recognition, image recognition, object recognition, speech recognition, healthcare, marketing, automotive, retail, etc.

GoogLeNet, ResNet50, and EfficientNet-b0 implemented in Matlab were used for the analysis. The input image has dimensions of 224 × 224 × 3 for GoogLeNet, ResNet50, and EfficientNet-b0. The above-mentioned convolutional neural networks can recognize 1000 object categories. GoogLeNet, available in Matlab software, consists of 144 layers. ResNet50, available in Matlab software, consists of 177 layers. EfficientNet-b0, available in Matlab software, consists of 290 layers. More information on GoogLeNet, ResNet50, and EfficientNet-b0 is described in the literature [19,20,21,22,23,24,25,26,27].

## 4. Results

An electric shaver was used as the source of the BLDC motor. The following states of the BLDC motor were analyzed: HB, 1FSB, 2FSB, and 3FSB. The PNID method was used to compute the area of the thermal image. Deep neural networks were used for the analysis of thermal images of the faulty shaft of the BLDC: GoogLeNet, ResNet50, and EfficientNet-b0. In total, 720 test thermal images (180 for each class) were analyzed to test the neural network. In total, 240 training thermal images (60 for each class) were used for the training of the neural network.

The parameters of neural networks were as follows: InitialLearnRate = 0.001, ValidationFrequency = 5, MaxEpochs = 500, MinBatchSize = 11, Solver—sgdm (stochastic gradient descent with momentum). The efficiency of recognition (*E*) for one state of the BLDC is defined as follows (1):

(1) $$E_{}=\;100\%\;*\;(X1)\;/\;(X2),\;$$

where *X*1 is the number of recognized test images and *X*2 is the number of all test images.

The arithmetic mean of *E* is expressed as follows (2):

(2) $$MeanE_{}=(E_{1}+E_{2}+E_{3}+E_{4})/4,$$

where *E*_1_ is *E* of HB, *E*_2_ is *E* of 1FSB, *E*_3_ is *E* of 2FSB, and *E*_4_ is *E* of 3FSB.

Table 1 shows the results of recognition for PNID and GoogLeNet, ResNet50, and EfficientNet-b0.

The obtained efficiency *E* and *MeanE* are equal to 100% (Table 1). PNID and GoogLeNet, ResNet50, and EfficientNet-b0 work very well for the analysis of thermal images of the BLDC motor. Table 2 shows the results of verification for GoogLeNet, ResNet50, and EfficientNet-b0.

The obtained efficiency *E* and *MeanE* are equal to 100% (Table 2) for all analyzed BLDC motors. GoogLeNet, ResNet50, and EfficientNet-b0 work very well for the analysis of thermal images.

## 5. Conclusions

The technique of thermographic fault diagnosis of the shaft of a BLDC motor is presented. BLDC motors of electric shavers were used. The following states of BLDC motors were analyzed: HB, 1FSB, 2FSB, and 3FSB. PNID, GoogLeNet, ResNet50, and EfficientNet-b0 were used for the analysis of thermal images of the faulty shaft of the BLDC motor. The shivering of the thermal imaging camera was in the range of 0–1.5 [m/s^2^]. The obtained thermal images were different. The obtained efficiency *E* and *MeanE* were equal to 100%. PNID, GoogLeNet, ResNet50, and EfficientNet-b0 work very well for the analysis of thermal images. Verification was carried out using GoogLeNet, ResNet50, and EfficientNet-b0.

This research proves that PNID and deep neural networks are very good for thermographic fault diagnosis. The technique based on PNID and deep neural networks can be used for industry, cars, trains, power tools, and electric devices. In the future, the thermographic fault diagnosis technique will be extended to use more thermal images, high-resolution thermal imaging cameras, new methods of data classification, more types of faults, and additional signal analyses such as acoustic and vibration analyses. There is also the suggestion to carry out an analysis for different values of shivering (vertical and horizontal). Measurements will be taken at the different camera–motor distances.

## Figures and Tables

**Table 1:** Results of recognition for PNID and GoogLeNet, ResNet50, and EfficientNet-b0.

State of the BLDC	*E* [%]
*E*_1_, HM	100
*E*_2_, 1FSB	100
*E*_3_, 2FSB	100
*E*_4_, 3FSB	100
	*MeanE* [%]
*MeanE*	100

**Table 2:** Results of verification for GoogLeNet, ResNet50, and EfficientNet-b0.

State of the BLDC	*E* [%]
*E*_1_, HM	100
*E*_2_, 1FSB	100
*E*_3_, 2FSB	100
*E*_4_, 3FSB	100
	*MeanE* [%]
*MeanE*	100

## Data Availability

Not applicable.
